# Construction and characterization of a saturated Tn-seq library of *Salmonella* Typhimurium ATCC 14028

**DOI:** 10.1128/MRA.00365-23

**Published:** 2023-10-05

**Authors:** Jérôme Trotereau, Romain Jouan, Delphine Naquin, Maxime Branger, Catherine Schouler, Philippe Velge, Peter Mergaert, Isabelle Virlogeux-Payant

**Affiliations:** 1 INRAE, ISP, Université de Tours, Nouzilly, France; 2 Université Paris-Saclay, CEA, CNRS, Institute for Integrative Biology of the Cell, Gif-sur-Yvette, France; University of Maryland School of Medicine, Baltimore, Maryland, USA

**Keywords:** *Salmonella* Typhimurium, Tn-seq, transposons, virulence plasmid, essential genes, TRANSIT, growth advantage, growth disadvantage

## Abstract

*Salmonella enterica* is an important foodborne pathogen. Here, we present the construction and characterization of a high-density transposon sequencing library of the *Salmonella* Typhimurium ATCC 14028 strain. Essential, advantageous, and disadvantageous genes for growth in rich culture medium were identified on the chromosome and the pSLT plasmid.

## ANNOUNCEMENT

*Salmonella* Typhimurium is a foodborne pathogen capable of colonizing a wide variety of hosts, including mammals, plants, and poultry, giving it many ways to reach humans at the end of the food chain. More than 430,000 *Salmonella* genomes are now publicly available, and one of the biggest challenges is to use this mass of data to assign a function to each gene. Bacterial transposon mutant libraries have long been used to identify genes important for bacterial survival, growth, or virulence. These methods were very useful but time-consuming; each mutant was screened one by one and did not allow an exhaustive study of the bacterial genome, including intergenic regions or gene domains. The recent combination of transposon mutagenesis with next-generation sequencing (NGS) has offered a solution to these problems. In a method called transposon sequencing (Tn-seq), the characterization by NGS of a pooled mutant library determines the essential genes in a genome, and the comparison of the mutant pool before and after selection in a condition of interest allows a quantitative analysis for each insertion expressed as a fitness score (1).

Construction of a Tn-seq library in *Salmonella* Typhimurium strain ATCC 14028 and library characterization were performed as described by Wallner et al. (2). Briefly, this strain, widely used in research labs and considered as a model strain, was conjugated with the *Escherichia coli* strain MFDpir [auxotroph for the synthesis of 2,6-diaminopimelic acid (DAP)] (3), harboring the plasmid pSAM_EC. After a 12-hour conjugation step, bacteria were carefully scraped, resuspended, and quantified on tryptic soy agar (TSA) plates supplemented with 50 µg/mL kanamycin without DAP, revealing the complexity of the transposon library of 1.34 × 10^6^ independent mutants. In parallel, 200 µL of the conjugation mixture was plated on about 400 TSA plates supplemented with 50 µg/mL kanamycin and without DAP to amplify the library. After overnight incubation at 37°C, colonies were pooled, aliquoted, and frozen at −80°C in 25% glycerol. DNA of the mutants was then extracted (Microbial DNA Mini Kit; Macherey-Nagel, Düren, Germany) and subjected to restriction, dephosphorylation, ligation of the adaptors, and PCR amplification as described in Wallner et al. (2) prior to sequencing using primers and adaptors shown in Table 1. Single-read sequencing (75 bp) with an Illumina NextSeq 550 was performed by the I2BC sequencing platform (CNRS; Gif-sur-Yvette, France), as well as demultiplexing, trimming, filtering, and mapping as described by Wallner et al. (2).

**TABLE 1:** Primers and adaptors used for library construction and verification^*a*^

Name	Sequence from 5′ to 3′	Type	Target (or function)	Reference
Bu070	TTCCCTACACGACGCTCTTCCGATCTGCAGC NN	Adaptor	Bu070 and Bu071 form an adaptor containing the barcode for sample multiplexing	(2)
Bu071	P- GCTGCAGATCGGAAGAGCGTCGTGTAGGGAAAGAGT-P	Adaptor		
Bu069	*AATGATACGGCGACCACCGAGATCTACACTCT*TTCCCTACACGACGCTCTTCCGATCT	Primer	P5 primer for PCR enrichment	
Bu068	*CAAGCAGAAGACGGCATACGAGAT*AGACCGGGGACTTATCA**TCCAAC**CTGT	Primer	P7 primer for PCR enrichment	

^*a*^ P-, 5′ or 3′ phosphate; underlined sequence corresponds to the barcode; the sequence in bold is the site recognized by the MmeI enzyme; the sequence in italics corresponds to the sequence of the Illumina P5 or P7 primers.

Further analyses were carried out with TRANSIT V3.2.0 software (4) to determine essential genes and genes with insertion resulting in an advantage or disadvantage for bacterial growth. TRANSIT uses a Bayesian method based on the sequence length containing TA sites without insertion completed by a hidden Markov model (HMM), considering local differences in the read count.

**Fig 1:**
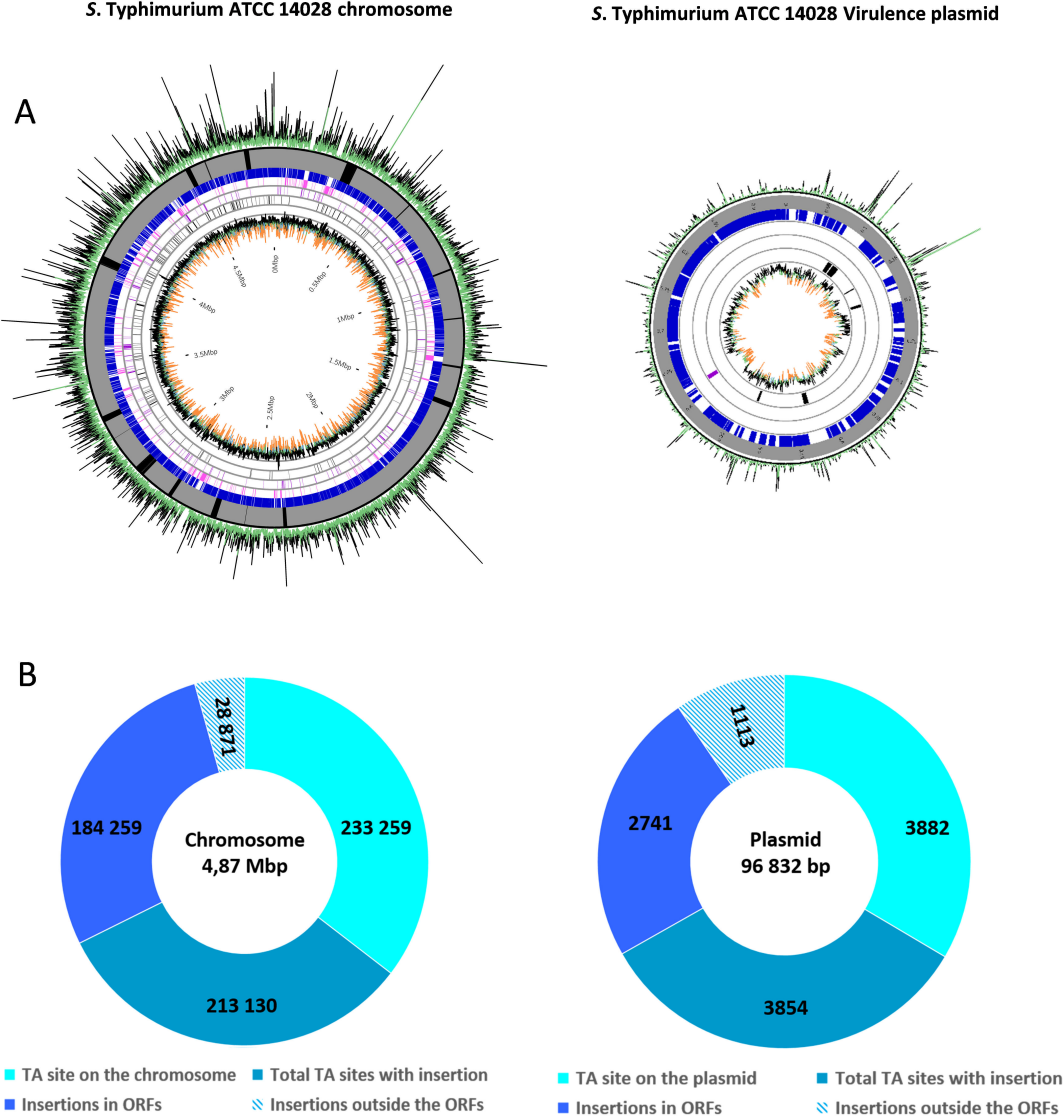
Circular visualization of the Tn-seq library in *S*. Typhimurium ATCC 14028 strain. (**A**) The chromosome and the virulence plasmid are saturated with insertions (green– and black peaks, below and above average, respectively). *Salmonella* pathogenicity islands on the chromosome are also saturated (SPI in black in the gray circle). Using TRANSIT software, we identified non-essential genes (blue lines), essential genes (pink lines), genes with insertion resulting in a growth defect (purple lines) and a growth advantage (black lines). The GC percentage of the genome is shown in black, green, and orange on the innermost circle (below, around, and above the average, respectively). (**B**) presents the number of TA sites and the number of insertions, as well as their distribution inside and outside the ORFs.

The genome of *S*. Typhimurium ATCC 14028 contains 4,626 open reading frames (ORFs) and 237,141 “TA” sites. Following sequencing, 6,351,674 reads were generated, out of which 3,425,806 could be uniquely aligned on the genome, corresponding to a coverage of about 15×. Our Tn-seq library is saturated with 213,130 and 3,854 TA insertion sites on the chromosome and the plasmid, respectively. No hot spots of transposon insertions or large gaps without insertions were identified (Fig. 1). Only five ORFs had no insertions, and 495 were identified as essential. Moreover, 253 and 244 chromosomal genes were identified as conferring either growth disadvantage or advantage to *S*. Typhimurium, respectively, when disrupted by the transposon. No essential genes were identified on pSLT, while one and seven growth disadvantage or advantage genes were identified on this virulence plasmid, respectively (data available here: https://doi.org/10.57745/GLMVCD). The Tn-seq library is available to the scientific community upon request to the corresponding author.

## Data Availability

The raw sequencing data are available on the ENA website under the following accession number: ERR10975930. Essential genes and genes with insertion conferring growth advantages or disadvantages are available at https://doi.org/10.57745/GLMVCD.

## References

[1] Cain AK , Barquist L , Goodman AL , Paulsen IT , Parkhill J , van Opijnen T . 2020. A decade of advances in transposon-insertion sequencing. Nat Rev Genet 21:526–540. doi:10.1038/s41576-020-0244-x 32533119 PMC7291929

[2] Wallner A , Busset N , Lachat J , Guigard L , King E , Rimbault I , Mergaert P , Béna G , Moulin L . 2022. Differential Genetic Strategies of Burkholderia vietnamiensis and Paraburkholderia kururiensis for Root Colonization of Oryza sativa subsp. japonica and O. sativa subsp. indica, as Revealed by Transposon Mutagenesis Sequencing. Appl Environ Microbiol 88:e0064222. doi:10.1128/aem.00642-22 35862731 PMC9317867

[3] Ferrières L , Hémery G , Nham T , Guérout A-M , Mazel D , Beloin C , Ghigo J-M . 2010. Silent mischief: bacteriophage Mu insertions contaminate products of Escherichia coli random mutagenesis performed using suicidal transposon delivery plasmids mobilized by broad-host-range RP4 conjugative machinery. J Bacteriol 192:6418–6427. doi:10.1128/JB.00621-10 20935093 PMC3008518

[4] DeJesus MA , Ambadipudi C , Baker R , Sassetti C , Ioerger TR . 2015. TRANSIT--a software tool for Himar1 TnSeq analysis. PLoS Comput Biol 11:e1004401. doi:10.1371/journal.pcbi.1004401 26447887 PMC4598096

